# Ictal dancing following right temporal seizure onset—Evidence for a distributed network

**DOI:** 10.1002/epd2.70218

**Published:** 2026-03-10

**Authors:** Leo Y. Zhang, German Garza‐Garcia, Alex Doyle, Irfan S. Sheikh

**Affiliations:** ^1^ University of Texas Southwestern Medical Center Dallas Texas USA; ^2^ Division of Epilepsy, Department of Neurology University of Texas Southwestern Medical Center Dallas Texas USA; ^3^ Peter O'Donnell Jr. Brain Institute University of Texas Southwestern Medical Center Dallas Texas USA

**Keywords:** syndrome: focal non‐idiopathic temporal (TLE), etiology: unknown, phenomenology: automatisms; hypermotor seizure, dancing, localization: temporal lobe (right); frontal lobe (right)

We report the case of a 44‐year‐old left‐handed male with a history of focal seizures occasionally evolving to bilateral tonic–clonic activity, managed with levetiracetam and lacosamide. During his phase 1 pre‐surgical evaluation, ictal EEG showed an abrupt onset of rhythmic 9–11 Hz alpha activity over the right temporal region that evolved into sharply contoured rhythmic theta and delta activity with rapid spread to the ipsilateral frontal, contralateral frontotemporal, and midline regions. Semiological correlation revealed right hand automatisms consisting of repetitive finger pointing followed by hyperkinetic “pop dancing” movements approximately 30–40 s after electrographical onset (Video 1).

**VIDEO 1:** Video‐EEG recording of ictal dancing semiology (LFF 1 Hz, HFF 70 Hz, Notch 60 Hz, Sensitivity 7 uV/mm). Bipolar montage shows first ictal change in the right frontotemporal region, maximal at F8‐T8 (00:50), with an abrupt onset of alpha activity followed by evolution in frequency, morphology, amplitude, and subsequent spread to bilateral hemispheric regions. Clinically, the patient experienced dancing semiology ~36 s following electrographic onset (01:26). Video content can be viewed at https://onlinelibrary.wiley.com/doi/10.1002/epd2.70218.

The onset pattern is similar to prior reports of dancing‐like semiology associated with temporal or frontal lobe epilepsy,^1^, ^2^, ^3^, ^4^ supporting the hypothesis that “ictal dancing” may arise from propagation within a distributed epileptogenic network. Notably, the absence of prior dancing‐like behavior and the presence of background music raise the possibility that environmental stimuli may have contributed to its emergence.

## AUTHOR CONTRIBUTIONS

L.Y.Z. reviewed and synthesized the clinical and EEG data, conducted the literature review, and drafted the manuscript. G.G.‐G. prepared the videos and figures and assisted with interpretation of the clinical and EEG findings. A.D. provided clinical expertise and contributed to content review. I.S. conceptualized the project, supervised its development, and provided critical revisions to both the manuscript and video. All authors approved the final version and agree to be accountable for the work.

## FUNDING INFORMATION

This work received no specific funding.

## CONFLICT OF INTEREST STATEMENT

L. Zhang, G. Garza‐Garcia, and A. Doyle report no disclosures related to this work. I. Sheikh is an associated editor for Epileptic Disorders Journal and is the assistant program leader of the Epileptic Disorders Internship Program.

## PATIENT CONSENT

Written informed consent was obtained from the patient for publication of the clinical video and EEG recording.

**Test Yourself**

1. Ictal dancing semiology has most often been associated with seizures originating in which brain regions?
  - Parietal lobe.
  - Temporal lobe.
  - Frontal lobe.
  - Both B and C.
2. Repetitive right hand finger pointing most strongly suggests:
  - Left temporal lobe involvement.
  - Right temporal lobe involvement.
  - Left frontal lobe involvement.
  - Right frontal lobe involvement.
3. The case suggests that ictal dancing may result from:
  - Seizure activity confined to a single cortical region.
  - Propagation within a distributed epileptogenic network.
  - Side effects of anti‐seizure medications.
  - Genetic predisposition to hyperkinetic movements.

*Answers may be found in the* *supporting information*.

## Supporting information


Figure S1

Data S1

Data S2

## Data Availability

No datasets were generated or analyzed for this educational submission. De‐identified clinical video and EEG recording are included within the article.
