# Supplementary material for: Ictal dancing following right temporal seizure onset—Evidence for a distributed network
Source: Epileptic Disord. 2026 Mar 10;28(4):1339–41. doi: 10.1002/epd2.70218 (PMC13499290; doi:10.1002/epd2.70218)
Supplement: Supplementary file 1 [file EPD2-28-1339-s001.docx]

Answers: (1) D (2) B (3) B.
