# Supplementary material for: Ictal dancing following right temporal seizure onset—Evidence for a distributed network
Source: Epileptic Disord. 2026 Mar 10;28(4):1339–41. doi: 10.1002/epd2.70218 (PMC13499290; doi:10.1002/epd2.70218)
Supplement: Supplementary file 2 [file EPD2-28-1339-s003.zip › epd270218-sup-0001-FigureS1@Supplementary Figure S1 - Legend.docx]

**Supplementary Figure S1**. Annotated ictal EEG screenshots from Video 1 depicting right temporal onset and propagation. (a) Average montage shows abrupt onset of low-amplitude 9-11 Hz alpha activity maximal at Fp2-F8 (red arrow), with more prominent delta activity seen over FT10 and further evolution into sharply contoured rhythmic theta and delta activity with rapid spread to ipsilateral frontal (Fp2-F8), midline (Fz-Cz), and contralateral frontotemporal (Fp1-F3) regions. (b) Laplacian montage shows abrupt onset of low-amplitude 1-2 Hz delta activity with superimposed fast activity maximal at F8-T8 (red arrow), evolving into rhythmic theta and delta activity with similar spread.
